# Delayed Hemopneumothorax and Multiple Rib Fractures Following Blunt Chest Trauma

**DOI:** 10.7759/cureus.115642

**Published:** 2026-09-02

**Authors:** Moawiah M Abdalrahman, Irena A Eftaiha, Sanaa M Abufares

**Affiliations:** 1 Department of General Surgery, Prince Hussein Bin Abdullah II Government Hospital, Amman, JOR

**Keywords:** blunt thoracic trauma, chest tube, delayed diagnosis, delayed hemopneumothorax, motor vehicle collision, respiratory distress, rib fractures, thoracostomy tube

## Abstract

Delayed hemopneumothorax is a surgical emergency, particularly following multiple rib fractures. We report the case of a middle-aged male presenting with chest pain due to a rare, late-onset development of traumatic hemopneumothorax. The patient initially presented following a motor vehicle collision (MVC) and was discharged from the emergency department (ED) with analgesics against medical advice. Vital signs were overall stable; initial imaging demonstrated right-sided apical pneumothorax with no signs of hemothorax and several right-sided rib fractures. Six days later, he returned with respiratory distress and severe chest pain, prompting further radiological investigations. Following the primary survey, the patient was managed definitively with tube thoracostomy placed via the triangle of safety and monitored with serial daily chest radiographs. The chest tube was removed on hospital day six, and the patient was discharged on day seven. He subsequently demonstrated complete resolution when evaluated at a 14-day follow-up appointment. This report illustrates a favorable clinical outcome achieved through timely management and emphasizes the importance of recognizing delayed hemopneumothorax as a potential complication of blunt thoracic trauma.

## Introduction

Multiple rib fractures are among the most common thoracic injuries following blunt trauma [1]. They are linked to increased mortality, pulmonary morbidity, and reduced quality of life. Displaced rib fractures can lacerate the lung and result in pneumothorax, hemothorax, or hemopneumothorax, which may be life-threatening [2]. A particularly challenging entity is the delayed presentation of hemopneumothorax, a complication characterized by the accumulation of blood and air within the pleural cavity hours, days, or even weeks after the initial injury [2,3].

While delayed hemothorax is a recognized complication of blunt thoracic trauma, particularly in patients with multiple rib fractures and high-energy mechanisms of injury, delayed hemopneumothorax following rib fractures remains less well characterized and has been infrequently reported in the literature. Unlike delayed hemothorax, which involves the accumulation of blood within the pleural cavity, delayed hemopneumothorax reflects the concurrent presence of blood and air within the pleural space, suggesting a combined pleural and pulmonary injury [2-4].

We report the case of a patient who developed delayed hemopneumothorax six days after sustaining multiple rib fractures following blunt thoracic trauma. This case highlights the importance of recognizing patients at increased risk for delayed pleural complications, distinguishing delayed hemopneumothorax from the more commonly reported delayed hemothorax, and emphasizing appropriate clinical reassessment and patient education after discharge.

## Case presentation

**Figure 1:**
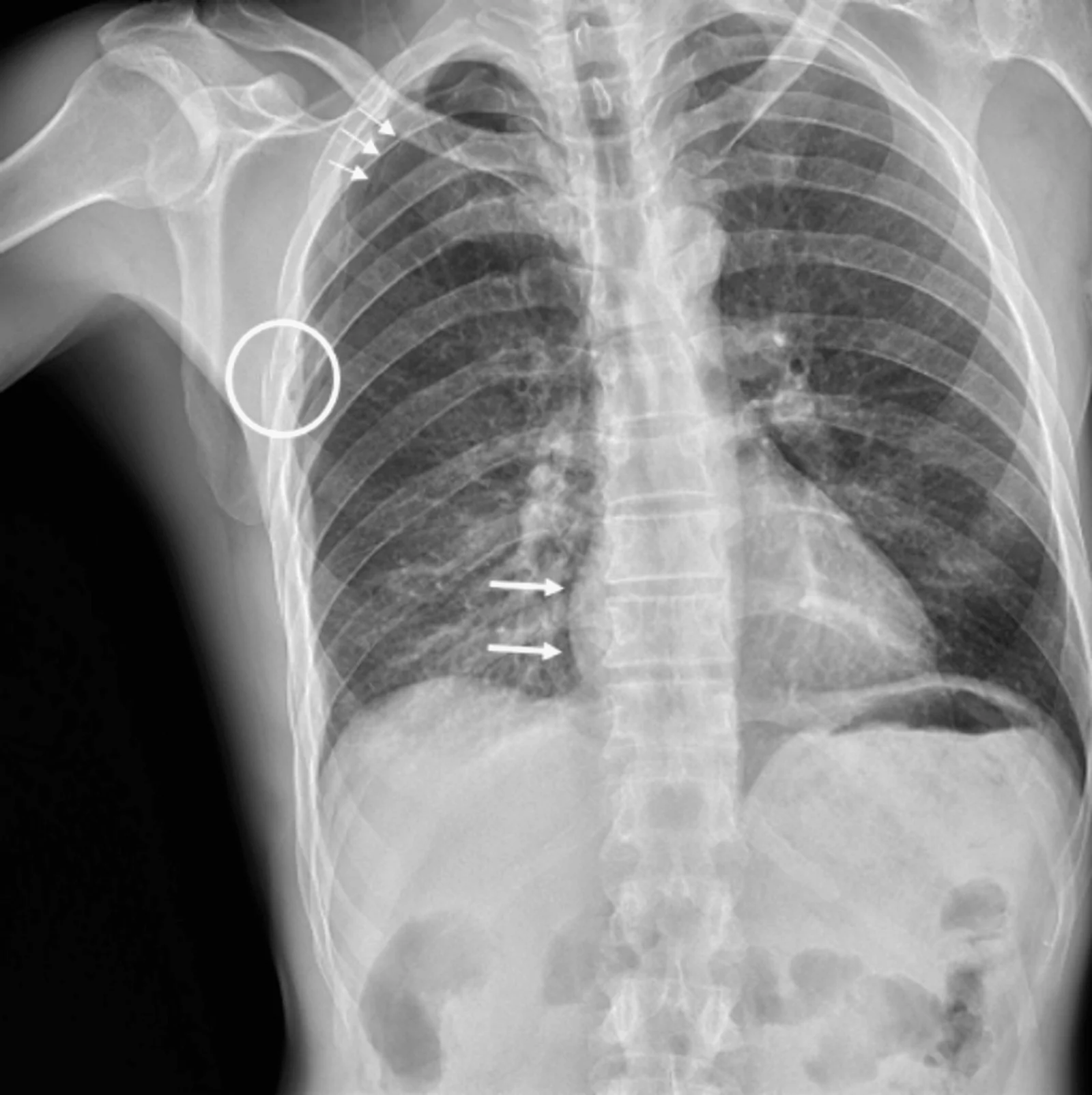
Initial anteroposterior (AP) chest radiograph obtained on the day of trauma. AP chest radiograph demonstrating a right-sided apical pneumothorax (upper white arrows) with no signs of hemothorax. The white circle highlights one of several right-sided rib fractures. Leftward displacement of the cardiac border indicates a mediastinal shift (lower white arrows).

A 52-year-old male patient with a history of chronic obstructive pulmonary disease, no known surgical history, and no drug or food allergies presented to the emergency department (ED) with mild chest pain after a motor vehicle collision (MVC) for evaluation and management. His vital signs were stable with oxygen saturation (SpO₂) of 95% on room air, blood pressure of 130/88 mmHg, and a heart rate of 104 beats per minute (bpm), which resolved spontaneously within a short period, and it was determined to be a stress-related response to the trauma. A comprehensive physical examination in the emergency department revealed clear breath sounds on auscultation and a symmetrical chest with adequate bilateral air entry. At the initial presentation, laboratory investigations were within normal limits, except for mild, clinically non-significant elevations in blood sugar (6.50 mmol/L) and blood urea (55.3 mg/dL) (Table 1). Initial diagnostic radiography revealed a right apical pneumothorax with multiple right-sided rib fractures with leftward displacement of the cardiac border, indicating a mediastinal shift (Figure 1). Further diagnostic workup, including additional imaging and inpatient monitoring, was recommended but refused by the patient, who was discharged against medical advice with oral analgesics. On day six post discharge, the patient returned with severe chest pain and respiratory distress, marked by labored breathing and tachypnea with tachycardia. His vital signs revealed a pulse of 115 bpm, a blood pressure of 132/76 mmHg, and an SpO₂ of 90% on room air. After initial stabilization in the ED and upon re-admission on post-injury day six, a repeat CBC was obtained (Table 1), and the patient was admitted for inpatient management and follow-up.

**Table 1:** Patient laboratory findings at initial presentation, re-admission, and discharge —: Indicates the parameter was not included in the targeted laboratory panel ordered

Parameter	Initial Presentation	Re-admission	Pre-Discharge	Reference Range	Unit
White Blood Cell Count	8.18	7.95	10.68	4.0-11.0	×10⁹/L
Red Blood Cell Count	4.78	4.67	5.09	3.5-5.5	×10¹²/L
Hemoglobin	14.7	14.6	15.6	13.5-18.0	g/dL
Hematocrit	41.7	41.9	47.5	40.0-51.0	%
Mean Corpuscular Volume	87.2	89.7	93.3	80.0-100.0	fL
Mean Corpuscular Hemoglobin	30.8	31.3	30.6	26.0-34.0	pg
Mean Corpuscular Hemoglobin Concentration	35.3	34.8	32.8	32.0-36.0	g/dL
Red Cell Distribution Width	12.4	12.7	12.8	11.0-16.0	%
Platelet Count	280	251	363	150-400	×10⁹/L
Mean Platelet Volume	10.7	10.6	11.0	6.0-11.0	fL
Plateletcrit	0.30	0.27	0.40	0.15-0.50	%
Platelet Distribution Width	12.2	12.8	13.2	11.0-18.0	%
Neutrophils Percentage	45.0	53.8	69.4	25.0-75.0	%
Lymphocytes Percentage	41.9	31.3	20.4	15.0-35.0	%
Monocytes Percentage	9.5	9.2	8.2	0.0-8.0	%
Eosinophils Percentage	3.4	5.4	—	1.0-6.0	%
Basophils Percentage	0.2	0.3	—	0.0-2.0	%
Fasting Blood Sugar	6.50	—	6.48	4.2-6.4	mmol/L
Urea	55.3	—	45.6	10.2-49.9	mg/dL
Creatinine	0.85	—	0.90	0.7-1.2	mg/dL
Sodium	138	—	137	135-148	mmol/L
Potassium	4.40	—	5.27	3.5-5.3	mmol/L
Calcium	—	—	9.16	8.5-10.5	mg/dL
Chloride	—	—	102	95-107	mmol/L

**Figure 2:**
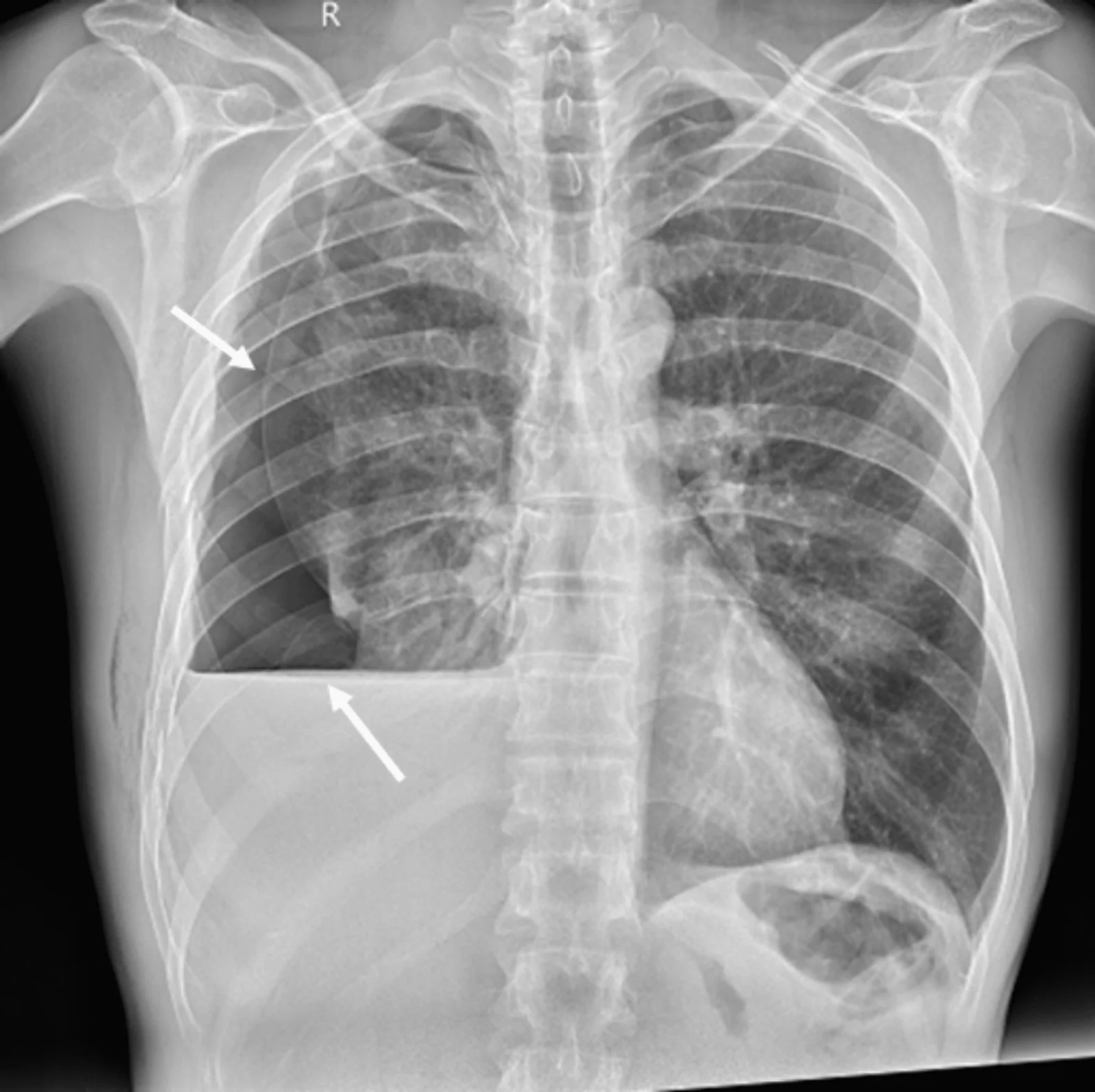
Upright anteroposterior chest radiograph obtained on day one of admission. Compared to the first radiograph, this chest radiograph demonstrates a right-sided pneumothorax with associated pleural fluid. The upper arrow indicates the visceral pleural edge of the collapsed right lung, while the lower arrow highlights the sharp, horizontal air-fluid level of the hydrothorax. The cardiac border is displaced to the left side, indicating a shift in the mediastinum.

On inspection, there was a noticeable asymmetrical chest expansion, with decreased movement on the right side during inspiration. On auscultation, breath sounds were diminished on the right side and normal on the left. Given his clinical deterioration, a repeat anteroposterior chest radiograph was obtained. The chest radiograph demonstrated right-sided pleural fluid with air, characterized by a distinct, straight horizontal air-fluid level in the lower-to-mid zone of the right hemithorax along with multiple fractures (Figure 2).

**Figure 3:**
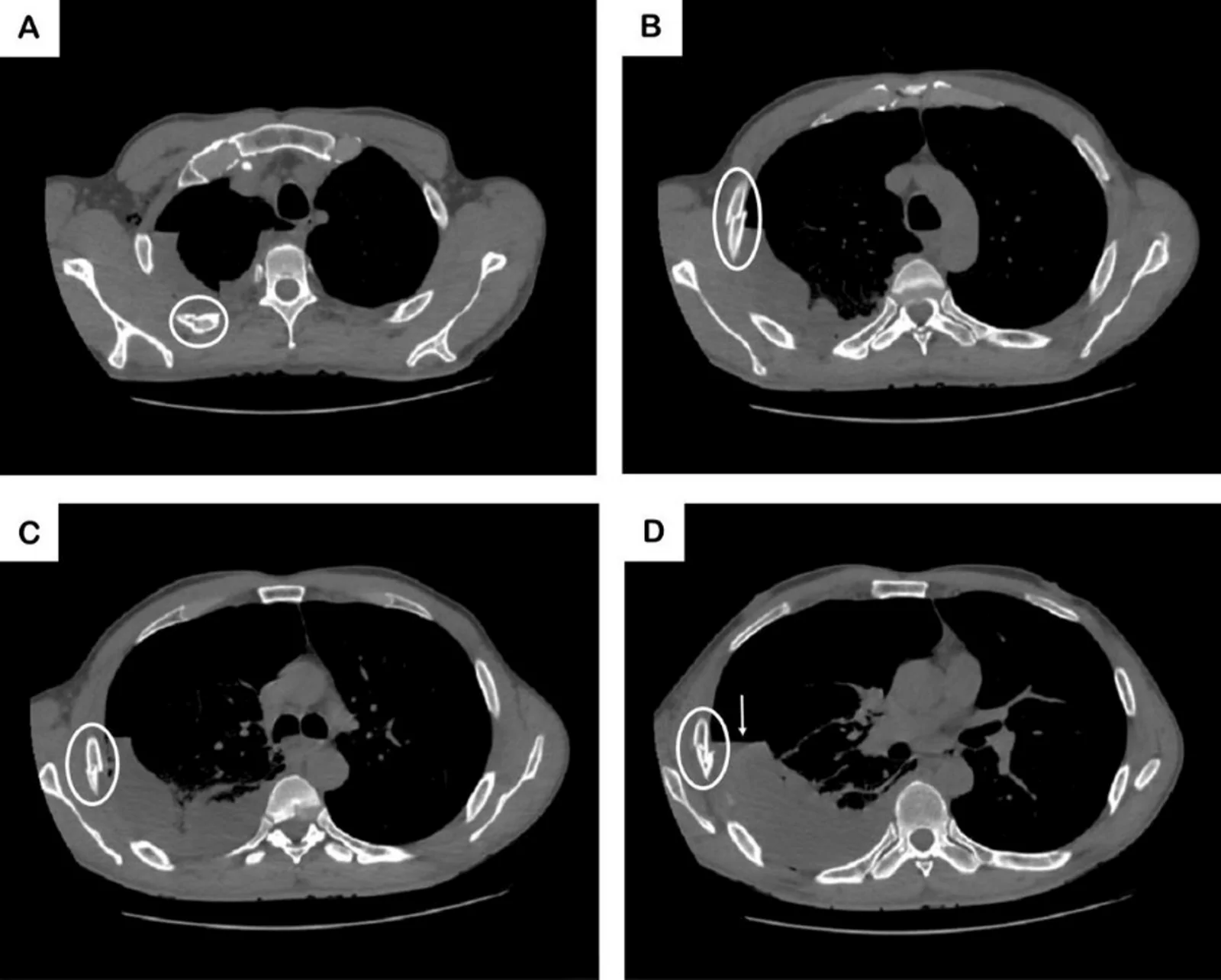
Non-contrast axial chest computed tomography (CT) scans (bone window), taken on day one of admission. (A) Scan demonstrates displaced posterior and lateral fractures of the right third rib (white circle). (B) Chest CT demonstrates a displaced lateral fracture of the right fourth rib (white circle). (C) Chest CT demonstrates a displaced lateral fracture of the right fifth rib (white circle). (D) Chest CT demonstrates a displaced lateral fracture of the right sixth rib (white circle), accompanied by a right-sided hydropneumothorax with a prominent flat air-fluid level (white arrow).

**Figure 4:**
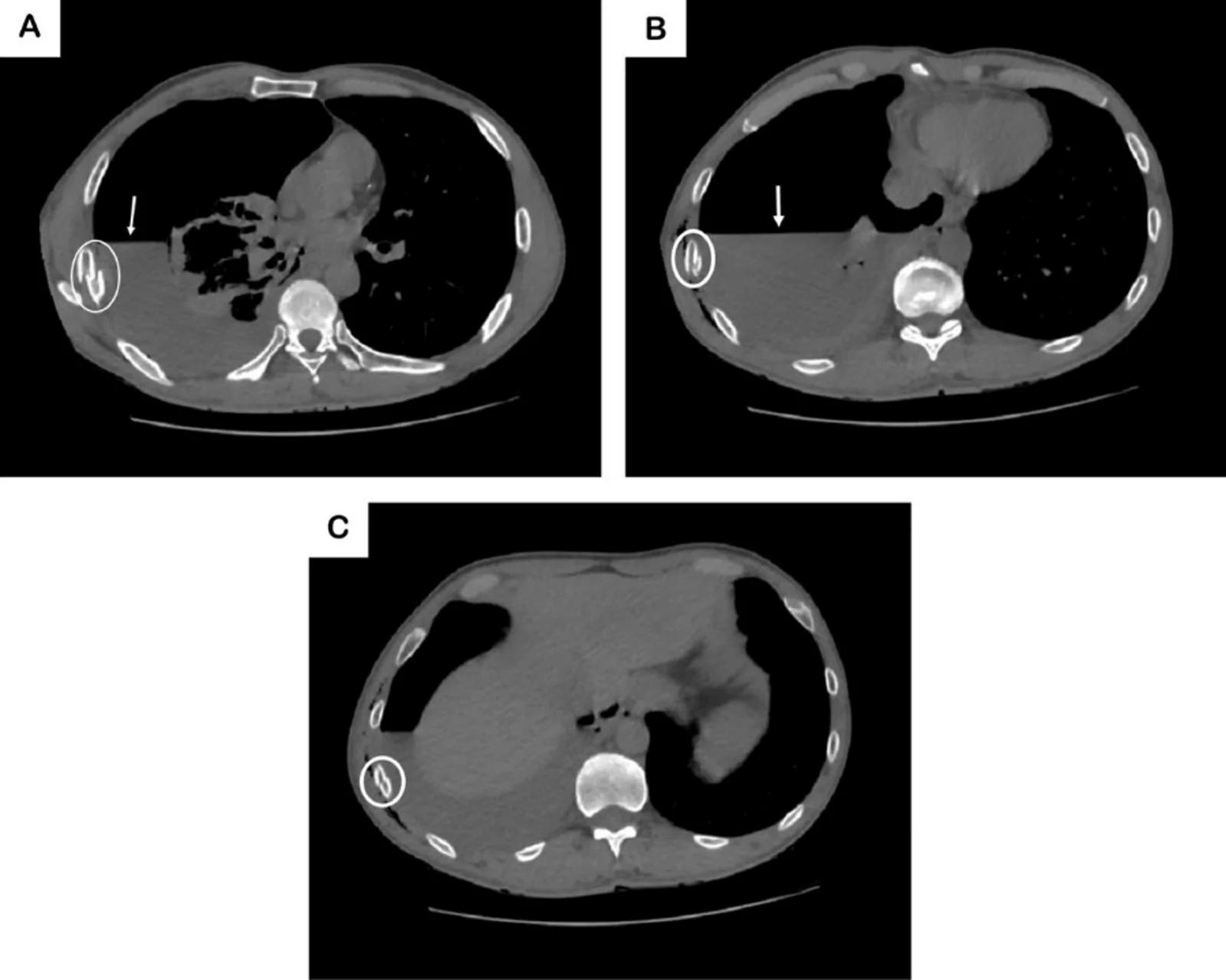
Non-contrast axial chest computed tomography (CT) scans (bone window), taken on day one of admission. (A) Chest CT demonstrates a lateral fracture of the right seventh rib (white circle), accompanied by a right-sided hydropneumothorax with a prominent flat air-fluid level (white arrow). (B) Chest CT demonstrates a displaced lateral fracture of the right eighth rib (white circle), accompanied by a right-sided hydropneumothorax with a prominent flat air-fluid level (white arrow). (C) Chest CT demonstrates a posterior fracture of the right ninth rib (white circle).

**Figure 5:**
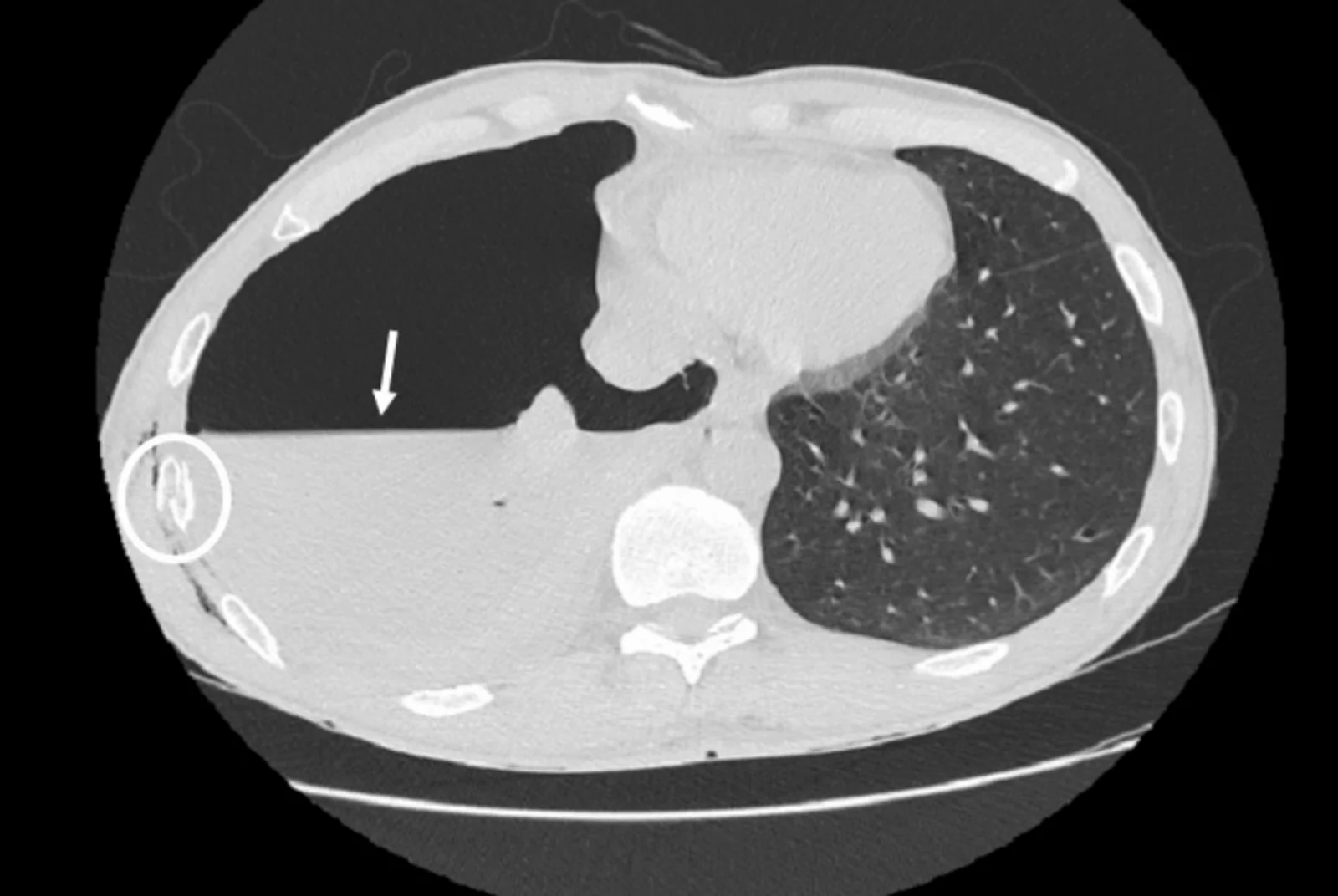
Axial computed tomography scan demonstrating a displaced rib fracture (lung window), taken on day one of admission. The scan reveals a displaced lateral right-sided rib fracture of the eighth rib (white circle) accompanied by a right-sided hydropneumothorax. The white arrow marks the flat air-fluid level.

**Figure 6:**
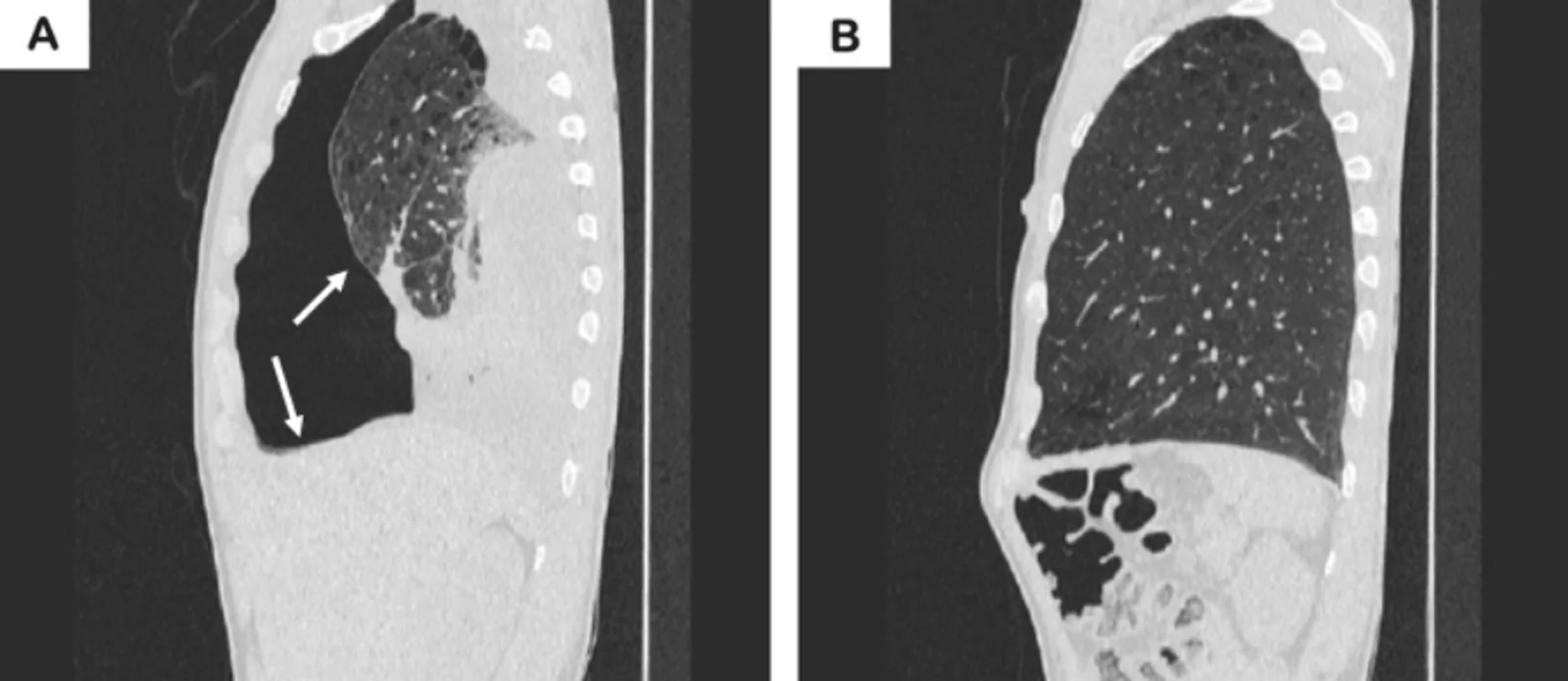
Sagittal chest computed tomography comparison, taken on day one of admission. (A) Sagittal slice of the affected hemithorax demonstrates a massive compressive pneumothorax (upper white arrow) displacing the collapsed right lung tissue, along with a significant dependent pleural effusion (lower white arrow). (B) Contralateral sagittal slice demonstrates the normal left side, fully expanded lung parenchyma, and a clear pleural space for anatomical comparison.

A subsequent computed tomography (CT) scan of the chest confirmed the right-sided hemopneumothorax and demonstrated eight distinct right-sided rib fractures across seven ribs: a displaced posterior fracture and a displaced lateral fracture of the third rib; a displaced lateral fracture of the fourth rib; a displaced lateral fracture of the fifth rib; a displaced lateral fracture of the sixth rib; a lateral fracture of the seventh rib; a displaced lateral fracture of the eighth rib; and a posterior fracture of the ninth rib (Figures 3-5). A sagittal reconstruction CT view was obtained to compare the affected right side with the contralateral normal left side (Figure 6).

The patient was given intravenous ceftriaxone (1 g every 12 hours) for prophylactic coverage and a single intramuscular dose of diclofenac for multimodal analgesia. After the imaging was reviewed by the thoracic surgery team, a tube thoracostomy was performed. Under local anesthesia, a 30 French, right-sided chest tube was inserted into the right pleural space via an open technique within the triangle of safety, bounded anteriorly by the lateral border of the pectoralis major, posteriorly by the anterior border of the latissimus dorsi, superiorly by the base of the axilla, and inferiorly by the fifth intercostal space. The tube was placed at the fifth intercostal space along the mid-to-anterior axillary line, dissecting directly over the superior border of the rib to avoid the intercostal neurovascular bundle, and was connected to an underwater seal drainage system.

**Figure 7:**
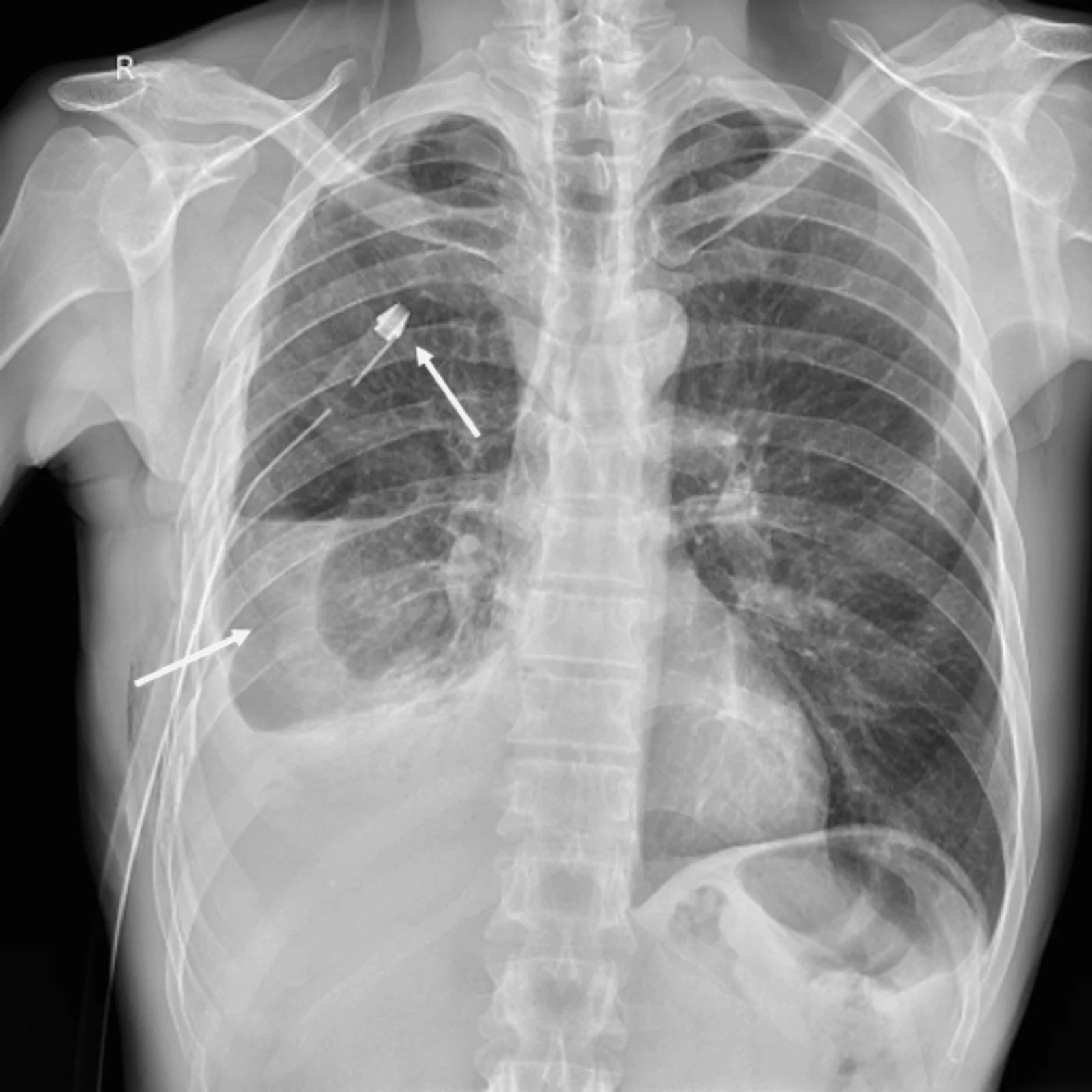
Post-intervention anteroposterior chest radiograph on day one of admission. The image demonstrates a newly inserted right-sided chest tube positioned within the upper pleural space (upper arrow). Compared to the previous radiograph, a residual air-fluid interface of the resolving hemopneumothorax is visible (lower arrow), demonstrating significant volume reduction and substantial re-expansion of the right lung parenchyma.

**Figure 8:**
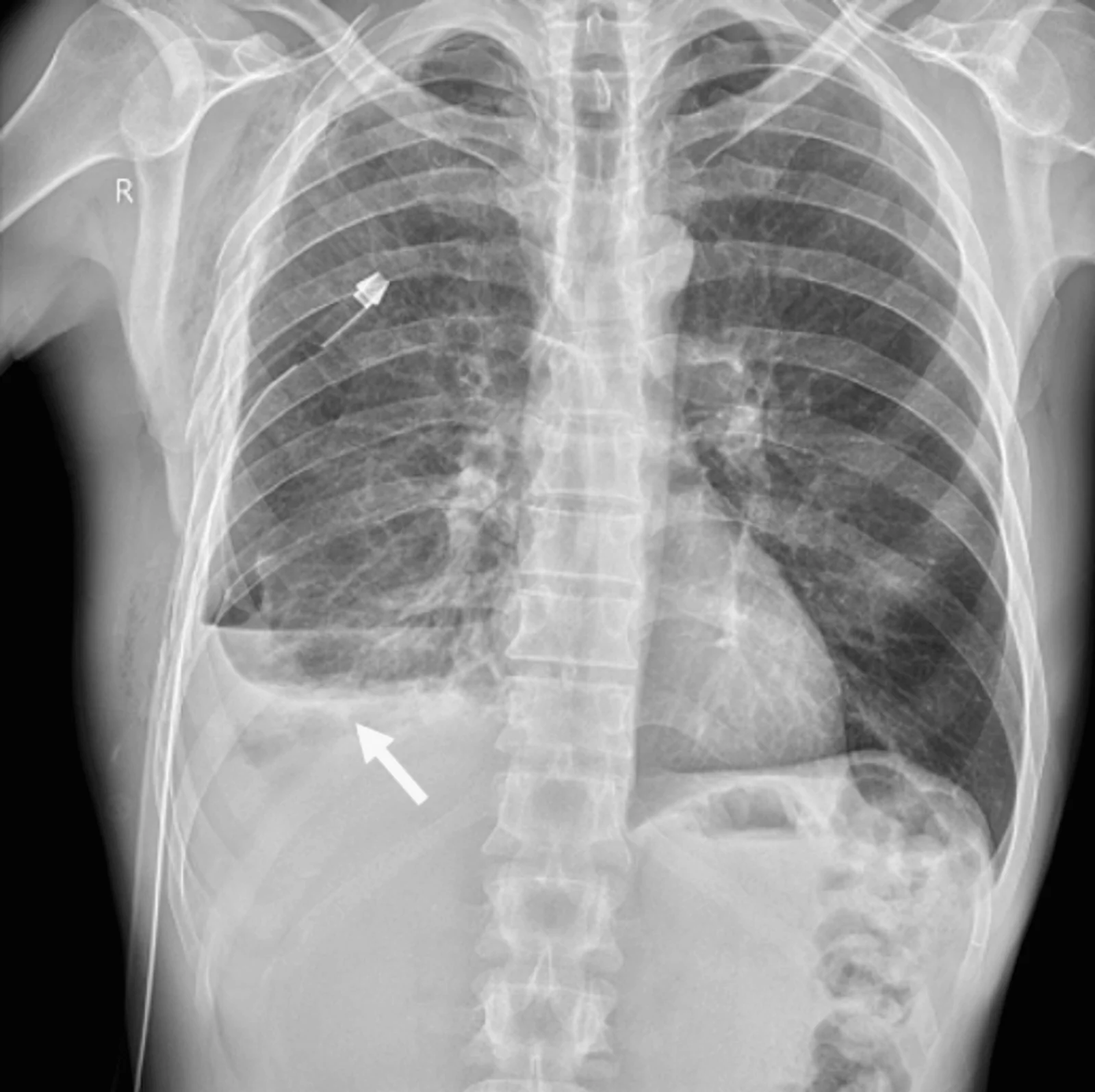
Follow-up anteroposterior chest radiograph on day two of admission. The image demonstrates ongoing clinical progress following right-sided chest tube placement, showing progressive re-expansion of the right lung parenchyma. Compared to the previous radiograph, the residual pleural fluid at the right lung base shows a further decrease in the volume of the hemothorax (white arrow).

**Figure 9:**
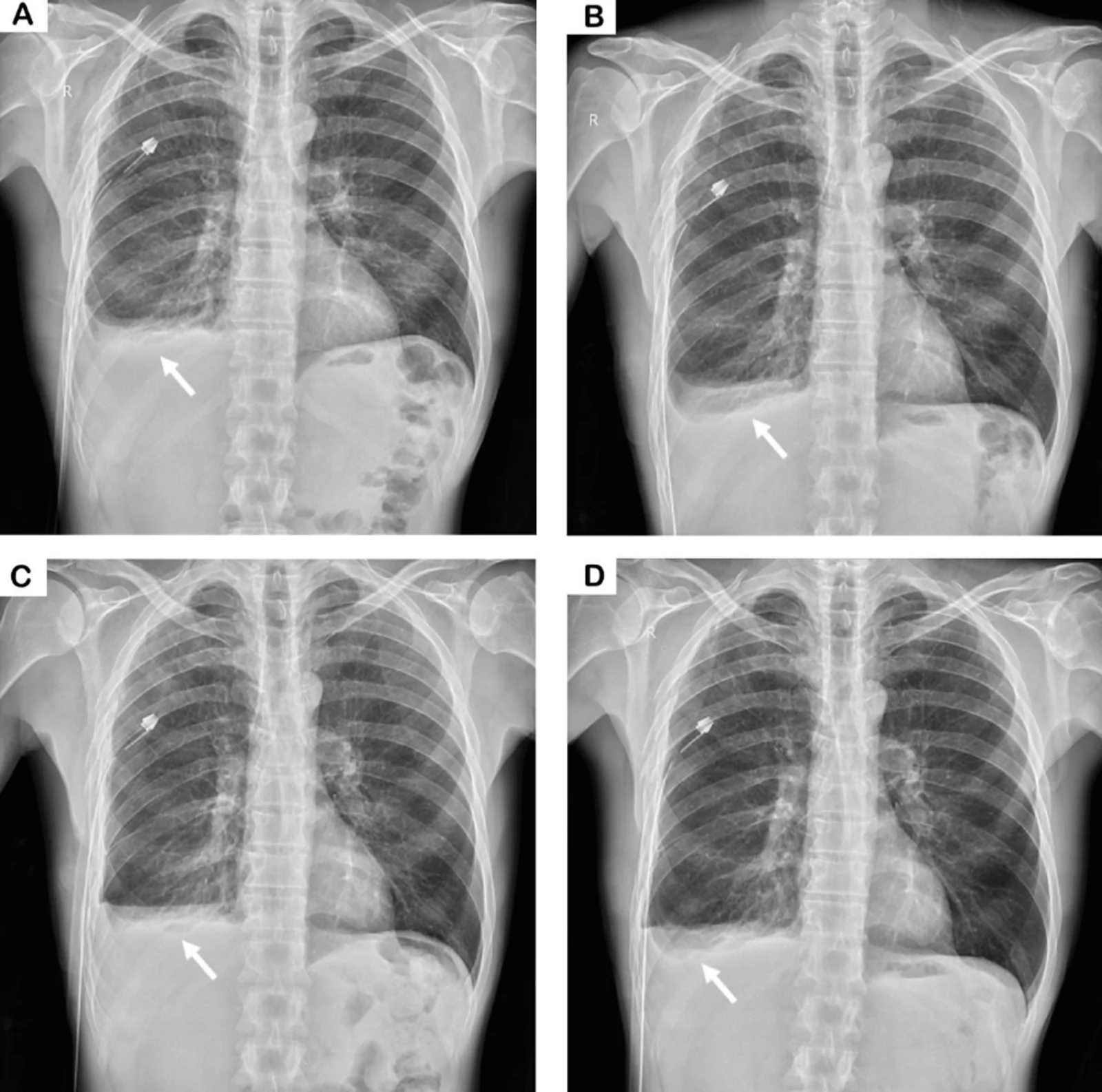
Serial daily follow-up anteroposterior chest radiographs. (A) Day three of admission: Post-drainage radiograph reveals the chest tube in situ with residual basilar fluid (white arrow). (B) Day four of admission: Notable decrease in pleural fluid volume (white arrow) with corresponding expansion of the right lung. (C) Day five of admission: Minor re-accumulation of dependent pleural fluid (white arrow) with a stable air-fluid interface. (D) Day six of admission: Final daily radiograph prior to chest tube removal demonstrates minimal residual pleural thickening at the costophrenic angle (white arrow) with near-complete resolution of the hemopneumothorax.

An immediate return of 200 mL of blood upon insertion confirmed the definitive diagnosis of delayed traumatic hemopneumothorax. A postprocedural chest radiograph confirmed appropriate tube positioning and partial re-expansion of the right lung (Figure 7). Following the procedure, the patient continued to receive standard antibiotic coverage, tailored analgesics, and maintenance fluids. As the patient did not prefer any surgical intervention, the surgical team managed the fractures conservatively. Serial daily chest radiographs were performed to monitor the patient's progress (Figure 8, Figures 9A-9D). There was no persistent air leak throughout the admission period, and no further active blood loss was observed beyond the initial 200 mL drainage.

**Figure 10:**
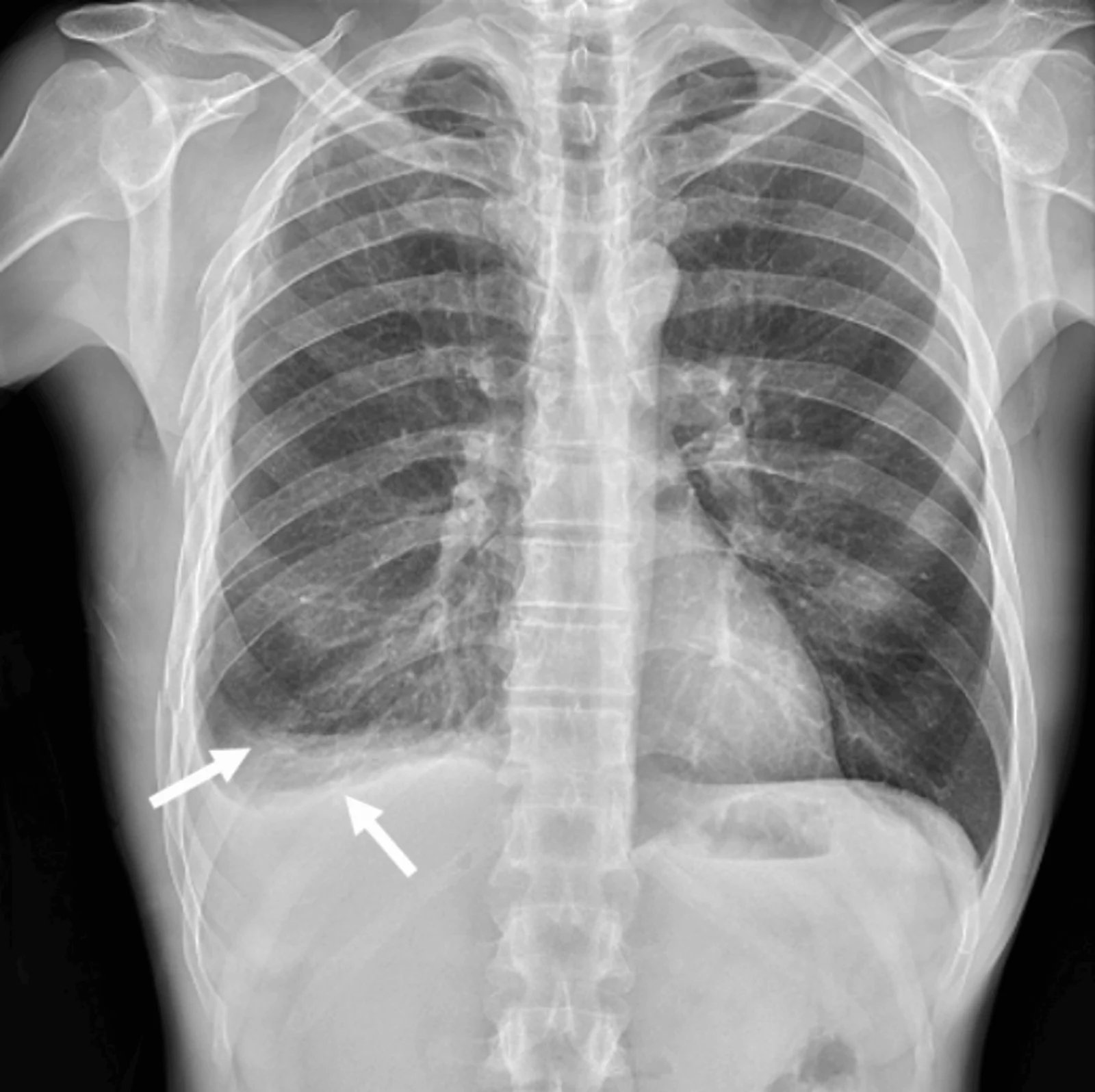
Anteroposterior chest radiograph obtained post-chest tube removal on day six of admission. An upright frontal chest radiograph obtained immediately following the removal of the right-sided chest tube. Compared to the previous radiograph, the lateral white arrow highlights blunting of the right costophrenic angle and localized pleural thickening, while the medial white arrow points to a small residual subpulmonic fluid collection. The lungs are otherwise well-expanded with no significant residual lateral or apical pneumothorax.

**Figure 11:**
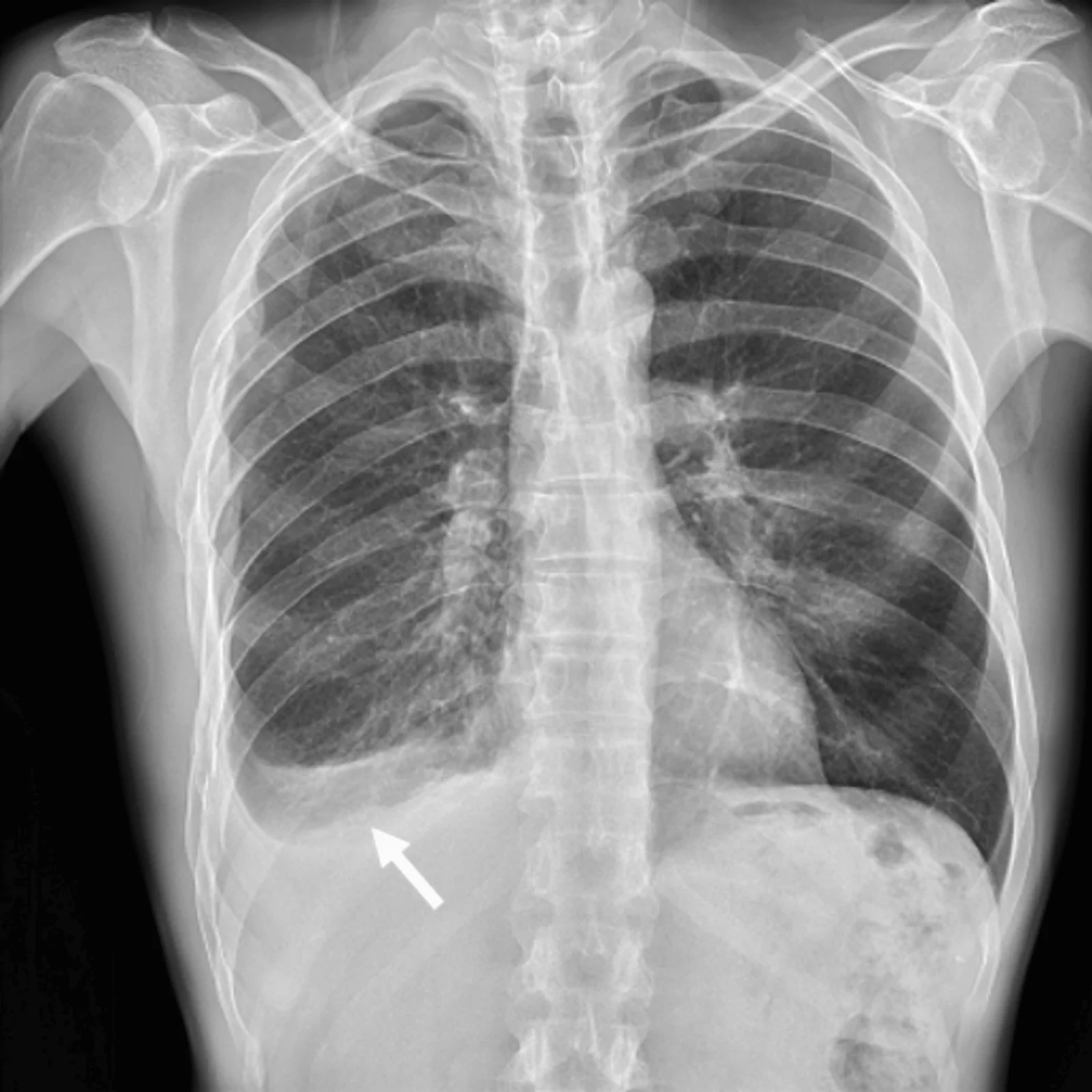
Final pre-discharge anteroposterior chest radiograph on day seven of admission. An upright chest radiograph obtained prior to hospital discharge demonstrates stable findings. Compared to previous radiographs, there is residual right-sided pleural thickening and trace fluid at the right lung base (white arrow) with blunting of the right costophrenic angle, and no evidence of recurrent hemopneumothorax.

**Figure 12:**
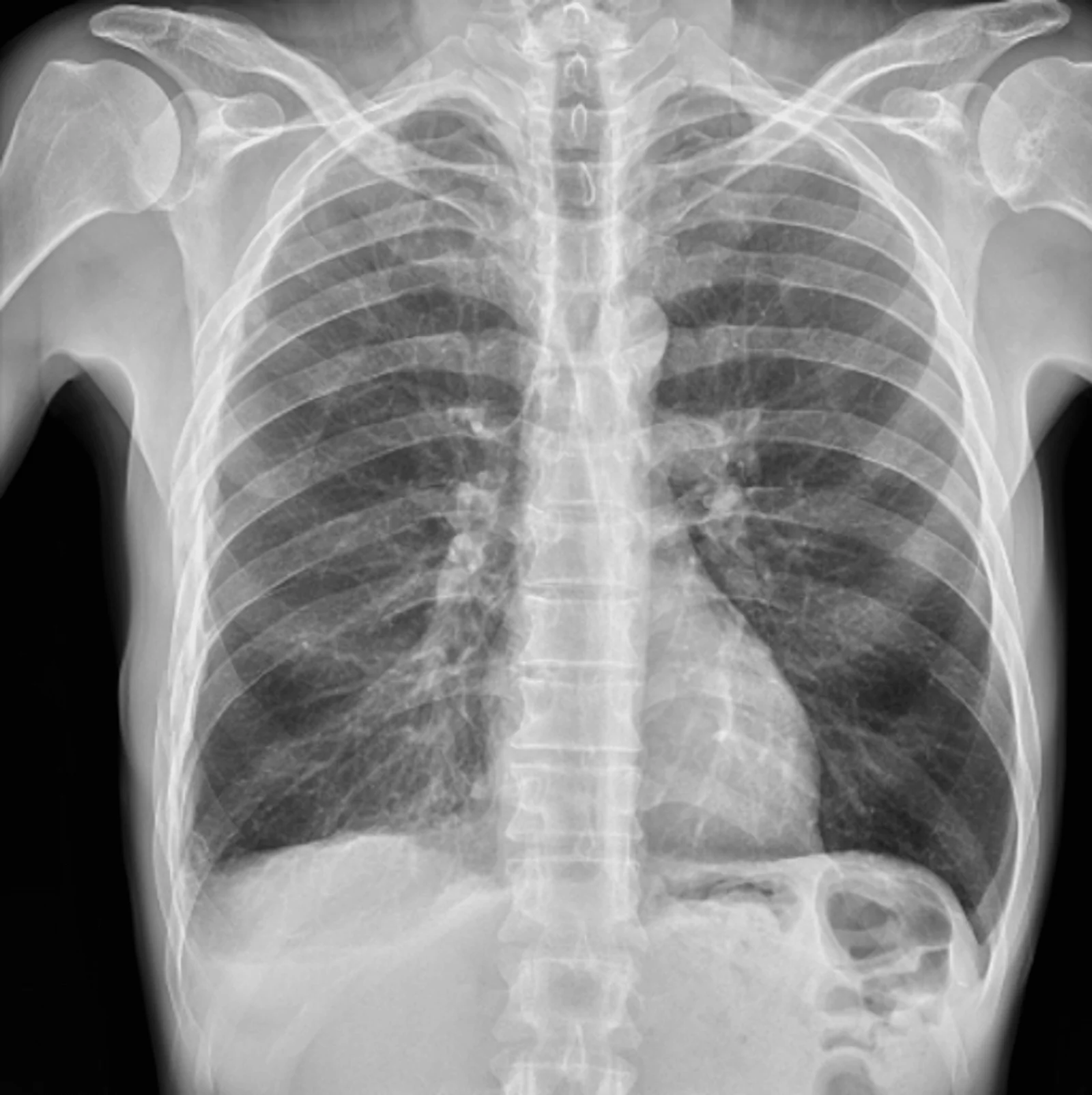
Anteroposterior chest radiograph at two-week post-discharge outpatient follow-up. An upright chest radiograph obtained during the patient's two-week outpatient follow-up visit demonstrates complete resolution of the right-sided hemopneumothorax, with clear lung fields and blunting of the right costophrenic angle.

Following clinical stabilization and definitive intervention, pre-discharge laboratory evaluation demonstrated reassuring cellular and metabolic indices (Table 1). The chest tube was removed on hospital day six after meeting standard removal criteria: absence of an air leak for over 24 hours, near-complete resolution of the hemopneumothorax, progressive lung re-expansion, and stable respiratory status. Post-removal chest radiography confirmed continued bilateral lung expansion (Figure 10). The patient was discharged on hospital day seven in stable condition (Figure 11), with instructions for outpatient follow-up. At his two-week follow-up appointment, the patient remained clinically stable and asymptomatic, with a chest radiograph demonstrating visible blunting of the right costophrenic angle and fully expanded lungs bilaterally (Figure 12).

## Discussion

Delayed hemopneumothorax is an uncommon but potentially serious complication of blunt thoracic trauma. Although delayed hemothorax is a recognized complication following blunt chest trauma, delayed hemopneumothorax is less frequently described in the literature. In our patient, a right-sided hemopneumothorax developed six days after a high-impact MVC associated with eight right-sided rib fractures across seven ribs. The timing of presentation is consistent with previous reports describing delayed hemothorax during the first week following injury [4-6].

The reported incidence of delayed hemothorax varies among studies according to the population and follow-up period. In a large retrospective study of patients with traumatic rib fractures, delayed hemothorax requiring readmission within 30 days occurred in a small proportion of patients [5]. However, the incidence of delayed hemopneumothorax specifically remains less well established, as the available literature predominantly describes delayed hemothorax rather than the combined accumulation of blood and air. Recent evidence has identified the number and displacement of rib fractures and the presence of pleural injury as factors associated with delayed hemothorax [7].

Rib fractures may injure the adjacent pleura, lung parenchyma, or intercostal vessels and may consequently result in pneumothorax, hemothorax, or hemopneumothorax [1-3]. CT may provide additional information regarding rib fractures and their characteristics when compared with initial imaging assessment [8]. This case demonstrated eight distinct right-sided rib fractures across seven ribs on CT scans performed at readmission. A proposed mechanism of delayed hemothorax is secondary injury to the pleura or intercostal vessels by fractured rib segments, with respiratory movement, coughing, or physical activity potentially contributing to delayed bleeding [2]. This may account for the development of pleural complications several days after the initial trauma.

Patients with delayed hemopneumothorax may present with dyspnea, chest pain, hypoxemia, and decreased breath sounds as blood and air accumulate within the pleural cavity [4,9]. In our patient, the delayed presentation was associated with acute respiratory distress, labored breathing, and decreased breath sounds on the right side. Chest radiography at readmission demonstrated a right-sided hemopneumothorax, and subsequent computed tomography demonstrated the pleural collection and multi-level rib fractures.

Management depends on the patient’s clinical condition and the extent of the pleural collection. Tube thoracostomy is an established treatment for clinically significant traumatic hemothorax and pneumothorax, allowing evacuation of blood and air, lung re-expansion, and monitoring of ongoing drainage [10]. In our patient, a right-sided 30-French chest tube was inserted under local anesthesia and connected to an underwater seal drainage system. Approximately 200 mL of blood was immediately drained. The patient was subsequently monitored clinically and with serial chest radiographs, which demonstrated progressive improvement of the hemopneumothorax. The chest tube was removed after clinical and radiographic improvement, and no additional surgical intervention was made upon the patient’s request.

Surgical management may be required in patients with ongoing significant intrathoracic bleeding or failure of tube thoracostomy to adequately control the hemothorax [10]. In this case, the patient remained clinically stable following tube thoracostomy and recovered without additional surgical treatment.

The literature specifically addressing delayed hemopneumothorax remains limited, with available evidence consisting predominantly of case reports and retrospective studies [5,6,9]. Most published data concern delayed hemothorax, and the available incidence estimates therefore cannot be directly extrapolated to delayed hemopneumothorax. This case adds to the limited literature by describing the delayed development of a right-sided hemopneumothorax six days after blunt thoracic trauma in a patient with multiple rib fractures. It also highlights the importance of reassessment when new respiratory symptoms develop following chest trauma.

## Conclusions

This case report emphasizes clinical recognition and management of delayed hemopneumothorax following blunt thoracic trauma with multiple displaced rib fractures. Given the risk of late-onset pleural complications, prolonged clinical vigilance and a high index of suspicion are essential when managing high-energy MVC casualties.

Patient care should incorporate a risk-based follow-up strategy, meticulous initial imaging review, and explicit discharge instructions outlining red-flag respiratory symptoms, particularly when patients decline initial observation or further inpatient evaluation. A structured outpatient follow-up protocol combining clinical examination, focused laboratory assessment, and interval chest radiography ensures timely identification of evolving pathology while balancing diagnostic yield against unnecessary serial radiation exposure.
